# Enhanced Bone Marrow Aspirate Concentrate (BMAC) Preparation Strategy in the Management of Chondromalacia Patella: A Case Report

**DOI:** 10.7759/cureus.59321

**Published:** 2024-04-29

**Authors:** Soundharya V, Arthi R, Hari Haran, Suresh Kumar I, Sahayaraj James

**Affiliations:** 1 Transfusion Medicine, Saveetha Medical College and Hospital, Saveetha Institute of Medical and Technical Sciences, Chennai, IND

**Keywords:** cd34+, bone marrow aspirate concentrate, regenerative therapy, growth factors, chondromalacia patella

## Abstract

Chondromalacia patellae (CMP) is a widespread cause of patellofemoral pain syndrome (PFPS), which manifests as anterior knee pain and functional limitations. Current treatments frequently fail to give long-term relief, necessitating the exploration of new therapeutic techniques. Recent research has demonstrated the efficacy of Bone Marrow Aspirate Concentrate (BMAC) therapy, which utilizes the regeneration characteristics of mesenchymal stem cells (MSCs) and growth factors. We present the case of a 36-year-old male patient with Grade III CMP who was resistant to conservative treatment but was successfully treated with BMAC therapy. Detailed methods for BMAC preparation, such as double centrifugation and growth factor analysis, are presented. At six and 12 weeks after therapy, the patient showed significant improvements in pain and functional results, as well as enhanced levels of growth factors and CD34+ cells in the BMAC. This study provides insights into the regeneration potential of BMAC therapy and highlights its promising role in managing chondral abnormalities. Larger clinical trials and standardization of BMAC preparation procedures are necessary for establishing its effectiveness and consistency as a standard treatment approach for CMP.

## Introduction

Chondromalacia patellae (CMP), also known as cartilaginous softening and fibrillation of patellar bone cartilage, is one of the potential causes of patellofemoral pain syndrome (PFPS). PFPS is characterized by anterior knee pain (AKP) and accounts for 10-25% of all visits between the age group of 18 to 45 years to physical therapy clinics [1]. Currently, there is no definitive cure for cartilaginous softening (e.g., CMP), posing a significant therapeutic challenge. Non-steroidal anti-inflammatory drugs (NSAIDs) may reduce pain in the short term, but pain does not improve after three months. Therapeutic ultrasound appears not to have a clinically important effect on pain relief for patients with PFPS. However, a few recent studies have demonstrated the feasibility of cartilage healing using mesenchymal stem cells (MSCs) [2]. Although several surgical approaches have been developed in the past decade to treat symptomatic patellofemoral cartilage defects, a gold standard treatment has not yet been determined [3]. Since the bone marrow itself is a source of MSCs, providing a cell population capable of chondrogenesis and various growth factors stimulating cartilage repair, an additional application of a bone marrow aspirate (BMA) to the procedure of marrow stimulation has been studied recently in the medical field. Moreover, the bone marrow clot creates a three-dimensional (3D) environment that promotes MSC chondrogenesis [4,5]. Bone Marrow Aspirate Concentrate (BMAC) is a concentrate of regenerative stem cells (autologous) containing mononuclear cells, thrombocytes, colony-forming unit (CFU)-fibroblasts, CD34+ and CD31-CD45-CD90+CD73+CD105+ cells [6]. They have a self-renewal potential generating various cell types, hematopoietic cells, fibroblastic reticular cells, and bone. BMAC therapy, which contains pluripotent mesenchymal stem cells (MSCs) and growth factors, has emerged as a promising intervention for regenerating cartilage tissue and alleviating symptoms. This method eliminates the need for an initial cartilage sample and subsequent chondrocyte cell cultivation, resulting in significant cost savings for the overall process [7].

We report a case of chondromalacia patella in a 36-year-old male with excruciating pain over the right knee, successfully treated with BMAC therapy. Presently, there is a lack of universally accepted standardized protocol for BMAC preparation. Our focus in this study lies in refining BMAC preparation methods to yield a superior product with improved clinical outcomes.

## Case presentation

**Figure 1:**
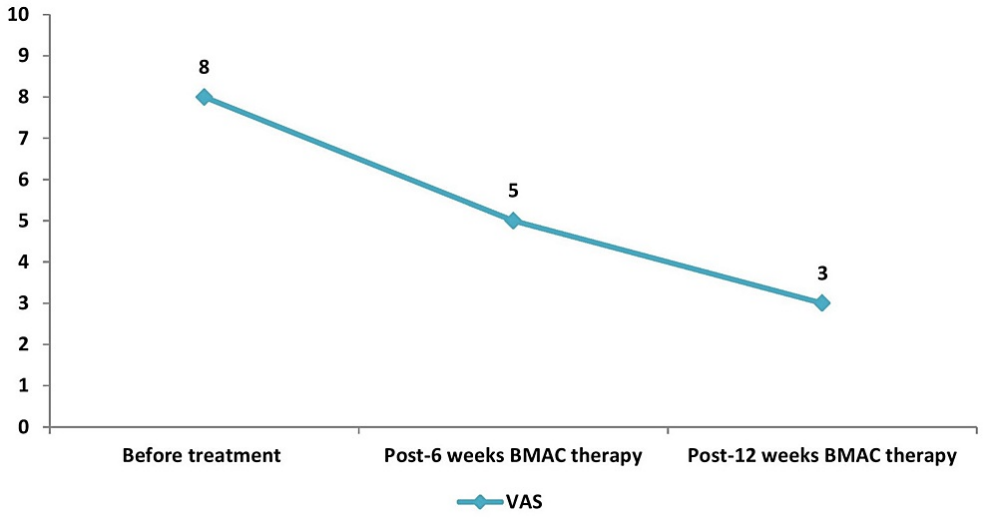
VAS score VAS: Visual analogue scale

**Figure 2:**
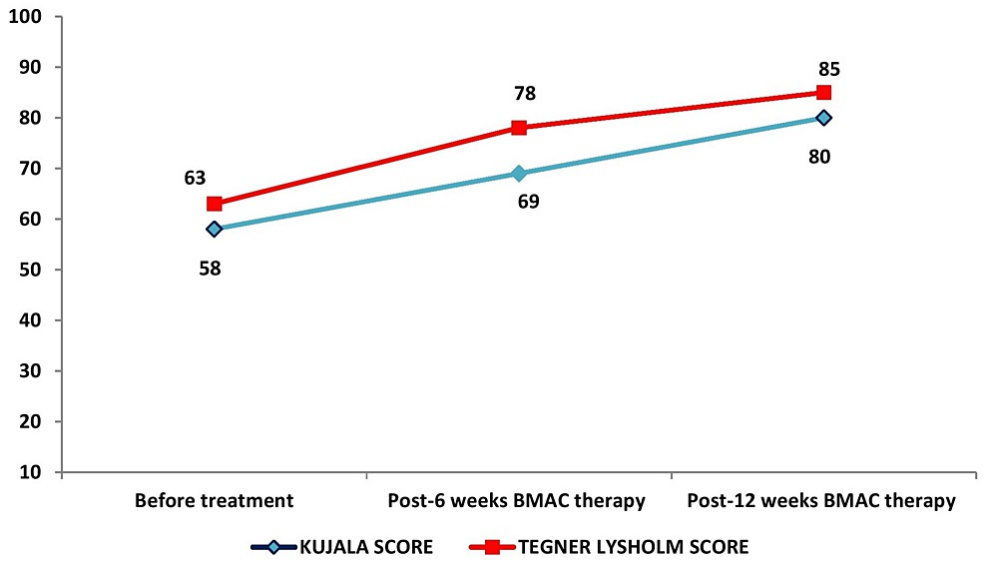
Kujala and Tegner Lysholm knee scoring scales

A 36-year-old male, who is a Laborer performing strenuous weightlifting duties, presented with a history of right anterior knee pain (AKP) exceeding one year. The patient developed AKP as a result of a trauma, particularly a fall over a year ago. He reported complaints of excruciating pain over the right knee for 1.5 years, insidious in onset and gradually progressive, hampering his daily physical activities. He also presented with a limited range of motion along with a giving away sensation of the right knee for four months. Following clinical (VAS: 8/10), functional assessment (Kujala score: 58/100), (Tegner Lysholm score: 63/100) [8], and radiological examination, the patient was diagnosed to have Grade III chondromalacia (modified Noye’s grading) [9] of the right patella. After a few weeks of non-steroidal anti-inflammatory drugs (NSAID) use in addition to physiotherapy (PT), the AKP reduced in intensity. However, about six months preceding the last visit, the patient had a relapse of AKP. Standing, walking, and exercising increased the discomfort, while rest alleviated symptoms. Unlike before, conservative approaches, such as physiotherapy and NSAIDs, did not appreciably relieve the discomfort. Therefore, BMAC administration was attempted for this patient to facilitate enhanced recovery. Upon obtaining informed consent, the patient underwent BMAC therapy utilizing autologous bone marrow-derived cells. The clinical assessment in chondromalacia patella was analysed using a visual analogue scale (VAS) (Figure 1), Kujala & Tegner Lysholm (Figure 2) knee scoring scales at six and 12 weeks.

**Figure 3:**
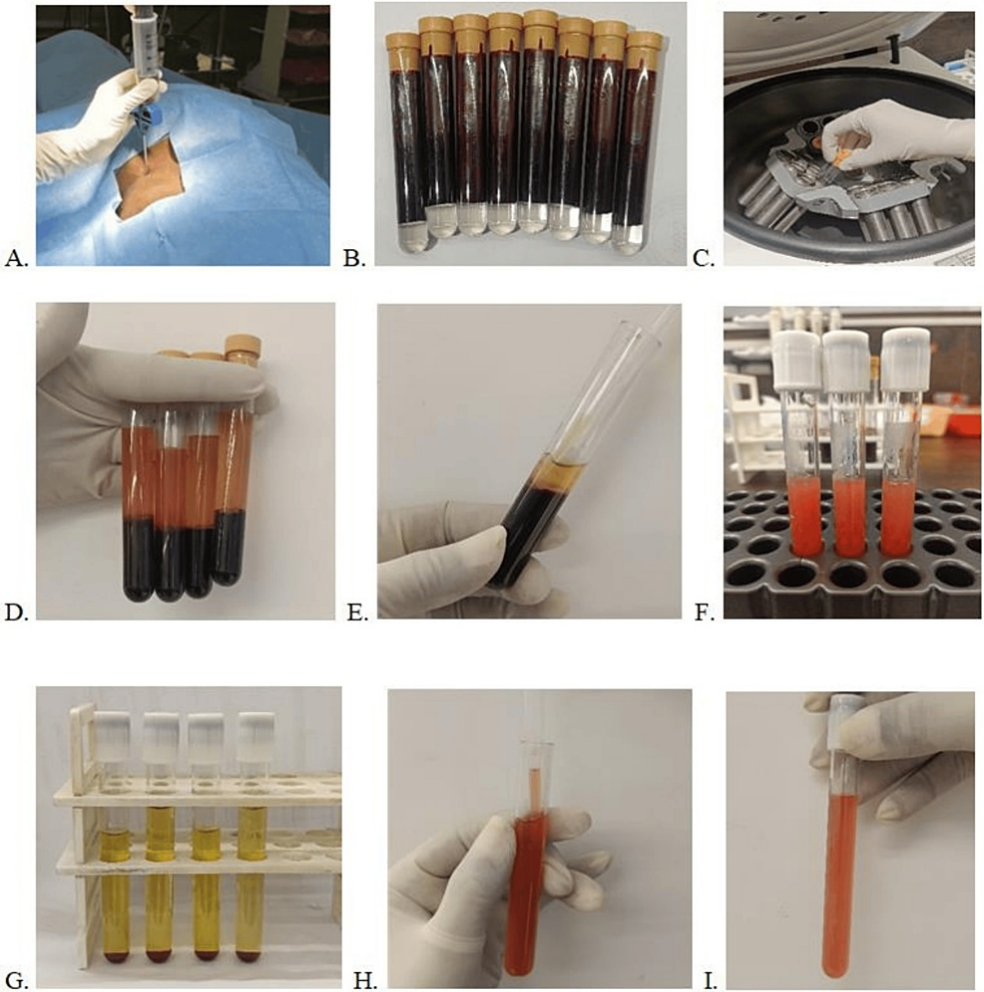
BMAC preparation procedure BMAC: Bone Marrow Aspirate Concentrate A. Positioning of bone marrow aspiration needle targeting postero-superior iliac spine, B. 80 ml of bone marrow aspirate (BMA) collected in sterile tubes, C. BMA subjected for first centrifugation, D. Separated red cells and supernatant, E. Removal of buffy coat with minimal red cells, F. BMA was transferred to separate sterile tubes, G. BMA post-second centrifugation, H. Resuspension of cell pellet for BMAC, I. Final volume of 10 ml BMAC.

Prior to the commencing of treatment, the patient endured a comprehensive evaluation, which included radiographic imaging, clinical assessment, and the use of standardized scoring systems such as the Kujala, Tegner Lysholm knee grading scales, and Visual Analog Scale (VAS). Strict aseptic procedures were followed in the operating theater (OT). Under ultrasound guidance, the postero-superior iliac crest was precisely targeted for bone marrow aspiration. Ten 8 ml tubes were collected from a 20-ml syringe that was previously coated with 2 ml of the anticoagulant heparin while maintaining sterility. Bone marrow aspirate (BMA) with a total volume of 80 mL was obtained. Double centrifugation was performed on the aspirate and an anticoagulant combination. The tubes were first centrifuged for 20 minutes at 40 g (720 rpm). Then, the buffy coat, the supernatant layer, and the few red cells were transferred into sterile containers and centrifuged for 10 minutes at 800 g (3200 rpm). Twenty percent of the supernatant was resuspended and the remaining 80% was discarded. In total, a volume of 10 ml of BMAC was obtained. In Figure 3, the steps involved in BMAC preparation are illustrated.

A representative sample was taken for the growth factor analysis and cell counting procedures. An automated hematology analyzer (SYSMEX XN 3100, Japan) was used to quantify the total nucleated count, platelets, and mononuclear cells. Flow cytometry (BD FACS CANTO II, USA) was used to quantify CD34+ cells. Following the instructions provided by the manufacturer, growth factors were measured using enzyme-linked immunosorbent assay (ELISA) kits (Elabscience Biotechnology Inc., USA) tailored to each growth factor. A single intra-articular BMAC injection was delivered using an 18-gauge needle and a sterile disposable 20-mL syringe containing 10 mL of BMAC, precisely targeted over the suprapatellar fossa of the right knee. The patient responded appropriately to a single BMAC administration session and experienced no adverse consequences immediately following administration. Three and six-month follow-up evaluations revealed persistent symptom alleviation without notable adverse events.

Follow-up evaluations at six and 12 weeks post-treatment, combined with two sessions of physiotherapy after BMAC administration, revealed significant improvements in pain severity (measured by VAS) and functional ability (assessed using Kujala and Tegner Lysholm knee scores). The VAS score dropped from 8 to 5, then to 3 (Figure 1), while the Kujala score increased from 58 to 69, then to 80 (Figure 2), and the Tegner Lysholm score rose from 63 to 78, then to 85 (Figure 2), compared to pre-treatment levels.

Quantitative analysis revealed a platelet count of 612.34 × 10^3^/μl, total nucleated cells of 104.51 × 10^3^/μl and mononuclear cells of 34.14 × 10^3^/μl (Table 1). Additionally, CD34+ count was determined to be 1.88% (refer to Table 1), with elevated levels of growth factors (basic fibroblast growth factor (b-FGF), platelet-derived growth factor (PDGF-BB), Vascular endothelial growth factor (VEGF), Transforming growth factor- beta 1 (TGF-𝛽1)) measured at 8.14 × 10^1^ pg/mL, 6.26 × 10^3^ pg/mL, 1.99 × 10^2^ pg/mL, and 1.89 × 10^4^ pg/mL, respectively (Table 2). These findings hold promising implications for tissue repair.

**Table 1:** Quantification of BMAC 𝜇l: Microliter; BMAC: Bone Marrow Aspirate Concentrate

Analyzed parameters	Values obtained
Platelet count (x 10^3^/𝜇l)	612.34
Total nucleated cells (x 10^3^/𝜇l)	104.51
Mononuclear cells (x 10^3^/𝜇l)	34.14
CD 34+ count (%)	1.88

**Table 2:** Growth factor concentrates in BMAC BMAC: Bone Marrow Aspirate Concentrate; b-FGF: Basic fibroblast growth factor; PDGF-BB: Platelet-derived growth factor; VEGF: Vascular endothelial growth factor; TGF-𝛽1: Transforming growth factor- beta 1; pg/mL: Picograms per milliliter.

Growth factors	Values obtained
b-FGF (× 10^1^ pg/mL)	8.14
PDGF-BB (× 10^3^ pg/mL)	6.26
VEGF (× 10^2^ pg/mL)	1.99
TGF-𝛽1 (× 10^4^ pg/mL)	1.89

## Discussion

Bone marrow aspirate concentrate (BMAC) has developed as an innovative treatment for chondral disease. BMAC is a source of growth factors, which are assumed to be essential due to their anabolic and anti-inflammatory actions, despite having a modest quantity of stem cells. BMAC treatment is a safe and rapidly developing practice, possibly due to its status as one of the FDA-approved categories for stem cell delivery. Recent studies showed positive outcomes, but the use of multiple outcome measures makes direct comparison difficult [10]. Brittberg et al. published the first report on autologous chondrocyte implantation (ACI) in 1994 [11]. They conducted ACI on 23 patients with full-thickness cartilage abnormalities in the knee joint. Two years following implantation, 14 of 16 patients with femoral condylar implantation had satisfactory outcomes. However, only two of the seven patellar implantations produced good or exceptional results [12].

A pure cohort with a patellofemoral cartilage defect showed significant improvements in all clinical scores, with a mean International Knee Documentation Committee (IKDC) increase of 43.7 points from 38.8 ± 19.2 to 82.5 ± 10.7 in a study by Gobbi et al [13]. Comparing BMAC in combination with a hyaluronic acid scaffold and matrix-induced autologous chondrocyte implantation (MACI) [14]. The latest research indicated that BMAC was more effective than hyaluronic acid (HA) and adipose-derived stem cells (ASC), demonstrating a treatment impact even at a 12-month follow-up. The ability of the pluripotent stem cells found in BMAC to encourage chondrogenesis and cartilage repair could be one reason [15,16]. BMAC was more beneficial for individuals with moderate-severe chondral abnormalities. Although augmentation techniques such as platelet-rich plasma (PRP) and adipose tissue transplants have been investigated, best practices are still unknown. To acquire further insight, randomized research involving control groups is required [3,10]. Although more research is necessary, b-FGF, which is found in BMAC in higher concentrations, is thought to be involved in the development and ongoing maintenance of cartilage. A recent study revealed the levels of growth factors in BMAC as follows: basic fibroblast growth factor (b-FGF) at 6.78 ± 5.87 × 10^1^ pg/ml, platelet-derived growth factor-BB (PDGF-BB) at 5.28 ± 2.57 × 10^3^ pg/ml, vascular endothelial growth factor (VEGF) at 1.76 ± 1.18 × 10^2^ pg/ml, and TGF-β1 at 1.56 ± 1.33 × 10^4^ pg/ml [6]. BMAC was made up of about 1.99% CD34+ cells. It has previously been demonstrated that CD34+ cells make up about 1% of all mononuclear cells [17]. Consistent with our results, other researchers have also observed a CD34+ fraction of nucleated cells ranging from 1.0% ± 0.2% in BMAC [18]. Skowroński and Rukta reported favorable clinical outcomes of BMAC with collagen membranes in large chondral lesions [19].

The limitations of BMAC injections were nonspecific and self-limiting. Symptoms such as pain, swelling, skin rash, itching, and aspirate site obstacles have been reported but generally resolved without any intervention. Oral and topical pain relievers (NSAIDs) were administered to alleviate the pain.

## Conclusions

This case underscores the potential of BMAC therapy as a promising treatment strategy for chondral defects, providing a minimally invasive option with satisfactory clinical outcomes. BMAC therapy promotes repair in chondral disease and additionally offers useful insights into regenerative therapy. Currently, there is no universally accepted standard method for preparing BMAC. Our approach to BMAC preparation resulted in significant improvement in a patient with chondromalacia patella. However, to validate the reliability and effectiveness of BMAC as a standard therapeutic strategy, larger clinical trials with extended follow-up and control groups are necessary.
